# Numerical calculation and experimental analysis of thermal environment in industrialized aquaculture facilities

**DOI:** 10.1371/journal.pone.0290449

**Published:** 2023-09-25

**Authors:** Zhipeng Yang, Desheng Li, Jiashuai Song, Encai Bao, Qiang Wang, Yue Qiu, Zhaoxue Wu

**Affiliations:** 1 College of Engineering, Anhui Agricultural University, Anhui Hefei, China; 2 Institute of Agricultural Facilities and Equipment, Jiangsu Academy of Agricultural Sciences, Key Laboratory of Facility Agricultural Engineering in the Middle and Lower Reaches of the Yangtze River, Ministry of Agriculture and Rural Affairs, Nanjing, China; Southwest Jiaotong University, CHINA

## Abstract

With the increasing market demand for high-quality aquatic products, the application of industrialized aquaculture facilities may get more attention. In order to improve the poor performance of thermal insulation, the accuracy of the numerical model was verified in this study through actual measured data. The model verification results shown that the average relative errors of the measured and calculated values of indoor air temperature, water temperature and roof inner surface temperature in the industrialized aquaculture workshop is within 2.5%, it suggested that the numerical calculation results are accurate. Furthermore, the thermal environment and thermal insulation performance of industrialized aquaculture facilities in winter were conducted based on the numerical calculations. After optimized the thermophysical parameters of the workshop enclosure structure, we found that the water body temperature could reach 21°C (which was close to the breeding temperature of grouper (Epinephelinae). Therefore, the numerical calculation method was further used to analyze the energy consumption of aquaculture water in January of a typical year in this area by heating to three constant temperatures (22, 25, and 28°C). When the aquaculture water was heated to the three constant temperature states, it needed to consume 8.56×10^5^, 1.02×10^6^ and 1.22×10^6^ MJ of energy respectively, which were equal to the amount of energy released by the complete combustion of 29.3, 35.1 and 41.8 t standard coal. Moreover, it is concluded that the artificial temperature increase in winter maintains the temperature in the range of 22~25°C to provide the highest heating efficiency. This conclusion can provide theoretical basis and application reference for industrialized aquaculture in winter.

## 1. Introduction

With global climate change and population growth, the risk of a food crisis for humanity has increased significantly [1–4]. To meet the ever-growing global demand for food due to stagnation in the fishing industry, aquaculture production faces significant pressure for expansion. In order to achieve sustainable development in aquaculture, it is crucial to substantially reduce the environmental impact of production [5]. Temperature is a critical environmental factor in the thermal environment of aquaculture, and it has a significant impact on the growth performance, reproduction, and disease susceptibility of aquatic organisms. Several researches [6–9] have demonstrated that temperature is a key factor determining the growth rate and yield of aquaculture species.

Extensive research has been conducted on the effects of temperature on the growth performance in aquaculture. Generally, temperature has a direct influence on various aspects of fish, such as metabolic rate, feed digestion and absorption, appetite, and energy utilization [10]. However, both excessively high and low temperatures can potentially result in growth limitations or abnormalities [11]. Furthermore, temperature also affects the gill respiration, oxygen delivery, and blood circulation in fish, thereby further influencing their growth performance [12–14]. Temperature also plays a crucial role in the reproduction of aquaculture species. It can influence gonadal development, secretion of sex hormones, quality and quantity of reproductive cells, thus affecting reproductive behavior, fertilization rate, egg development, and survival rate of larvae [15, 16]. High or low temperatures can potentially lead to delays or interruptions in the reproductive period, thereby negatively impacting population growth and sustainability. In addition, temperature is closely associated with the susceptibility of aquaculture species to diseases. Temperature can influence the immune function and disease resistance of cultured organisms, making them more prone to infections by pathogenic microorganisms [17]. Therefore, long-term or short-term exposure to suboptimal temperatures can impair the organism’s ability to resist pathogens, thereby compromising the overall health of the animals [18].

The design and layout of aquaculture facility structures are crucial for maintaining an optimal thermal environment. The insulation performance of the facility is the key factor [19, 20]. Rational selection and arrangement of insulation materials and facilities can reduce temperature differences between the interior and exterior of the facility, minimize heat transfer losses, and provide a more stable temperature environment. Several scholars have investigated how the properties of structure materials affect the thermal environment of facilities. In the winter of 1985, Tiwari [21] verified that combining a movable insulation covering system with the heat storage function of a concrete north wall could reduce heat loss in a greenhouse at night. Asan H [22–24] discovered that the thermal performance parameters of a wall had a significant impact on the delay time and decay factor. Furthermore, the influence of insulation layer thickness and material type on the time lag and lapse factor was studied, which revealed a profound influence of both factors on the time lag and lapse factor. Jelle B P et al. [25] investigated the existing thermal insulation materials and the application possibility of thermal insulation materials in the future. Some scholars [26] carried out a large number of experiments on the thermal conductivity of the existing thermal insulation materials to further reduce the uncertainty in the simulation process. However, the problem of energy utilization efficiency has always been a major issue that needs to be solved in industrialized aquaculture. The application of computational fluid dynamics (CFD) could provide a feasible technical mean to solve this problem. As early as 1991, Losordo et al. [27] proposed a mathematical model for simulating the thermal stratification of shallow aquaculture ponds, and established a dynamic mechanical model of pond water columns in discrete, fully mixed horizontal volume units. In order to minimize the energy demand of the industrialized recirculating aquaculture system (RAS). Singh et al. [28] established a stepwise steady-state thermal model to simulate the RAS thermal environment, and simulated various production scenarios. Jain et al. [29] established a transient analytical model for the study of more uniform greenhouse heating effects in aquaculture ponds in extreme winters, and selected a typical winter sunshine model for solution verification. The optimal design parameters are obtained by changing the influence of single structural parameters of the fish pond on the water temperature of the fish pond. Klemetson et al. [30] established the MAPT (Maintenance of Aquaculture Pond Temperatures) aquaculture pond temperature maintenance model, which can provide a convenient means of potential estimation in the absence of site data. Zhu et al. [31] established a GPS (Greenhouse pond systems) greenhouse pond system, which can be used to describe various heat transfer and temperature and humidity changes under different climatic conditions. Sarkar et al. [32] established a heat balance model for greenhouse ponds in the central Himalayas, which showed that passive greenhouse fish ponds with uniform spans can increase the indoor water temperature by 4.14°C compared with the water temperature of outdoor ponds.

Most of the above studies were aimed at the thermal environment research of planting facilities and the water temperature of outdoor ponds. The studies on thermal environment simulation inside aquaculture facilities are still scarce. In recent years [33–35], the problems of poor winter heating and high operational and running costs in aquaculture facilities remains unresolved. The innovation of this study lies in conducting experimental measurements on various components of aquaculture workshops and developing a numerical model for the structural analysis of these facilities. After validating the reliability of the numerical model, the heat loss of the aquaculture facilities was observed through the temperature cloud map. Subsequently, the thermal insulation performance of the maintenance structures was optimized using the numerical model, and the heat loss of each component in the cold winter environment was calculated. This research provides valuable insights for the design of maintenance structures in aquaculture facilities and the provision of heating during winter production in aquaculture facilities.

## 2. Numerical approach and boundary condition

### 2.1 Governing equation

The aquaculture facility was modeled as a steady-state, three-dimensional, incompressible turbulent flow process using the *k* − *ε* (realizable) turbulence model. The control equation was discretized using the finite volume method, and the pressure coupling equation was solved using the SIMPLE algorithm (semi-implicit method for pressure coupling equations). The numerical calculation of airflow was performed based on the principles of fluid mechanics conservation. The formula is as follows:

**Continuity equation**:

$$\frac{\partial \rho }{\partial t}+div\left(\rho \vec{v}\right)=0$$ (1)

**Momentum equation**:

$$\frac{\partial \left(\rho \vec{u}\right)}{\partial t}+div\left(\rho \vec{u}\vec{v}\right)=div\left(\mu grad\vec{u}\right)-\frac{\partial p}{\partial x}+S_{\vec{u}}$$ (2)

$$\frac{\partial \left(\rho \vec{v}\right)}{\partial t}+div\left(\rho \vec{v}\vec{v}\right)=div\left(\mu grad\vec{v}\right)-\frac{\partial p}{\partial y}+S_{\vec{v}}$$ (3)

$$\frac{\partial \left(\rho \vec{w}\right)}{\partial t}+div\left(\rho \vec{w}\vec{v}\right)=div\left(\mu grad\vec{w}\right)-\frac{\partial p}{\partial z}+S_{\vec{w}}$$ (4)

**Energy equation**:

$$\frac{\partial \left(\rho T\right)}{\partial t}+div\left(\rho \vec{v}T\right)=div\left(\frac{k}{cp}gradT\right)+\frac{S_{T}}{C_{p}}$$ (5)

Where *ρ* is the density, kg/m^3^; *t* is the time, s; $\vec{v}$ is the velocity victor; $\vec{u}$, $\vec{v}$, $\vec{w}$ are the components of the fluid particle velocity in the x, y, z directions respectively; *μ* is the viscosity coefficient; *S* is the source term; *T* is the temperature, °C; *k* is the thermal conductivity, W/(m·°C); *C*_*p*_ is the specific heat capacity, J/(kg·°C); *p* is the pressure, Pa.

**Realizable *k* − *ε* model**:

*k* equation:

$$\frac{\partial \left(\rho k\right)}{\partial t}+\frac{\partial \left(\rho ku_{i}\right)}{\partial x_{i}}=\frac{\partial }{\partial x_{j}}\left[\left(\mu +\frac{\mu_{i}}{\sigma_{k}}\right)\frac{\partial k}{\partial x_{j}}\right]+Gk-\rho \varepsilon$$ (6)

*ε* equation:

$$\frac{\partial \left(\rho \varepsilon \right)}{\partial t}+\frac{\partial \left(\rho k\varepsilon u_{i}\right)}{\partial x_{i}}=\frac{\partial }{\partial x_{j}}\left[\left(\mu +\frac{\mu_{i}}{\sigma \varepsilon }\right)\frac{\partial \varepsilon }{\partial x_{j}}\right]+\rho C_{1}S\varepsilon -\rho C_{2}\frac{\varepsilon^{2}}{k+\sqrt{v\varepsilon }}$$ (7)

Where *G*_*k*_ is the turbulent kinetic energy generated by the average velocity gradient; *C*_1_ = max[0.43, *η*/(*η* + 5)], *η* = *Sk*/*ε*, *σ*_*k*_ = 1.0, *σ*_*ε*_ = 1.2, *C*_1_ = 1.44, *C*_2_ = 1.9.

### 2.2 Geometric model

**Fig 1:**
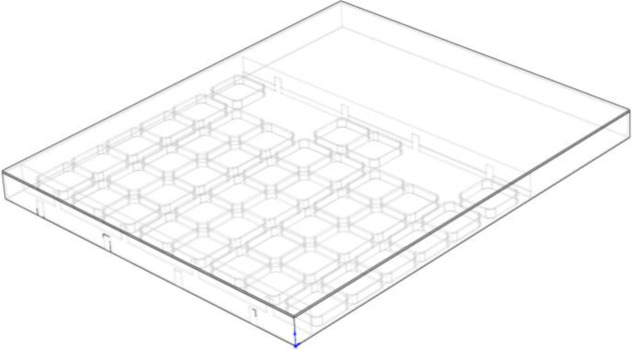
3D model.

To accurately predict the indoor temperature patterns, a 3D model of the aquaculture workshop was created using Solidworks at a 1:1 scale based on the dimensions of the experimental industrialized aquaculture workshop (Fig 1). The model was imported into ANSYS DesignModeler for further processing, such as splitting the model and defining boundary conditions. The geometric model consists of several domains, including the indoor air, equipment room air, artificial room air, pool solid, water body fluid, brick wall solid, and thermal insulation benzene board solid domains.

### 2.3 Meshing

Since the model involves a total of 1215 feature surfaces, its structural features are relatively complex. In order to improve the calculation accuracy, this study adopts the combined polyhedron and hexahedron grid type. Polyhedral grids are used in the fluid-solid boundary region, and hexahedral grids are used in the air domain. Firstly, the surface mesh is defined by ANSYS Mesh, and then import into Fluent Meshing to discretize the geometric area. The minimum unit length is defined as 5mm, and the number of grids generated after discretization is 1.892 million. The specific grid information is shown in Table 1.

**Table 1:** Grid information table.

Name	Number of grids	Maximum distortion
Air	1,366,000	0.89
Sink	97,900	0.83
Water	358,100	0.85
Insulation benzene board	52,800	0.84
Brick wall	175,800	0.86
Total	1,892,000	0.89

### 2.4 Boundary conditions

The maintenance structure outside the breeding workshop defines as the convective heat transfer boundary conditions, and the measured outdoor temperature is used as the simulation boundary. Assuming that the outdoor wind speed is maintained at 0.5 m/s, the convective heat transfer coefficient obtained is 7.6 W/(m^2^°C). The ground interface involved in the breeding workshop is defined as the heat flow boundary condition, and the set value is simulated by taking the heat flow at different times. The interface between the brick wall of the outer enclosure structure and the thermal insulation benzene board, and the interface between the indoor air and the maintenance structure define coupled heat conduction. Coupling heat conduction is defined at the interface between the brick wall of the outer protective structure and the thermal insulation benzene board, and at the interface between the indoor air and the maintenance structure. The simulation ignores the air domain between the artificial room and the equipment room, and the interface with the breeding area is defined as the temperature boundary of each area. The specific parameters are shown in Table 2.

**Table 2:** Physical characteristic parameters.

Material	Density (kg/m^3^)	Specific heat capacity (J/kg·°C)	Thermal Conductivity (W/m·°C)
Insulation benzene board	30	2414.8	0.041
Brick wall	1600	1051.1	0.5
Air	1.23	1006.43	0.0242
Water	998.2	4182	0.6

## 3. Materials and methods

### 3.1 Test site

**Fig 2:**
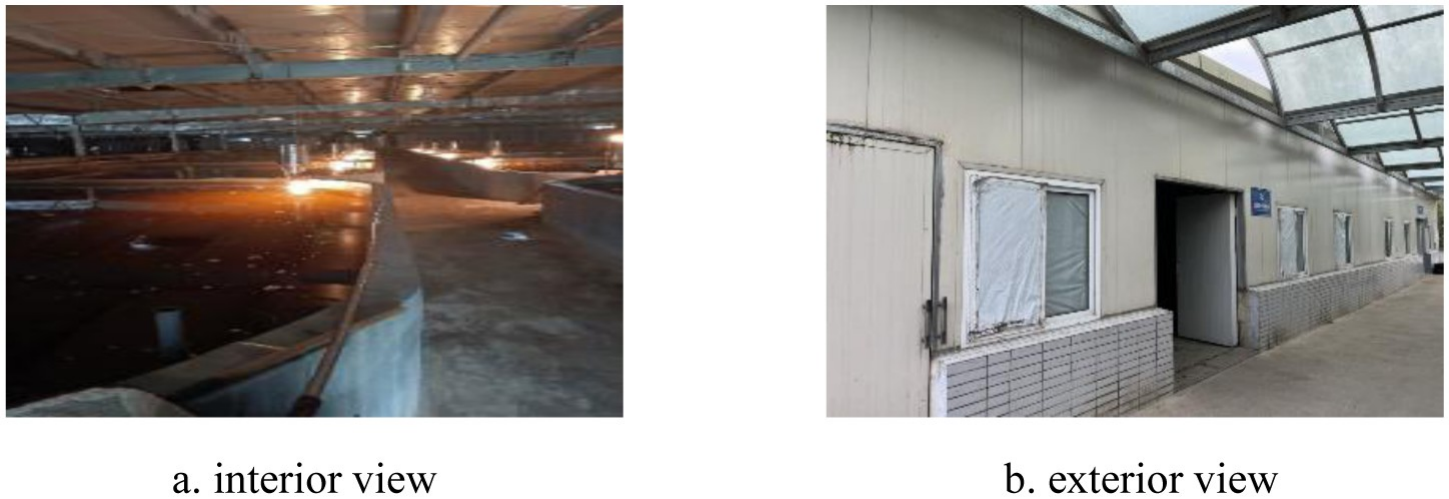
Workshop scene. a. Interior view, b. Exterior view.

The experimental workshop was a large-scale aquaculture workshop in Zhongyang Fugu Manor (120.90°E, 32.62°N) in Haian County, Nantong City. The interior and exterior views of the workshop were shown in Fig 2. The ridge of the aquaculture workshop was in the east-west direction. The length and width of the workshop were 67.2m×57.8m. The height of the ridge of the workshop was 3.5m, and there were four doors (0.9m×2.1m) on the west wall of the workshop, with a spacing of 13.2m. The maintenance structure around the workshop was a combination of brick walls and foam sandwich panels. The height of the brick walls was 0.8m. The top of the workshop was laid with plastic cloth, polystyrene foam and polyethylene film, with a thickness of 0.25m. The aquaculture system adopted high-density recirculating aquaculture technology, and the species cultured in the pond during the test period was grouper. During the test period (December 20, 2022 to January 10, 2023) the workshop was in a fully enclosed breeding state.

### 3.2 Test site

**Fig 3:**
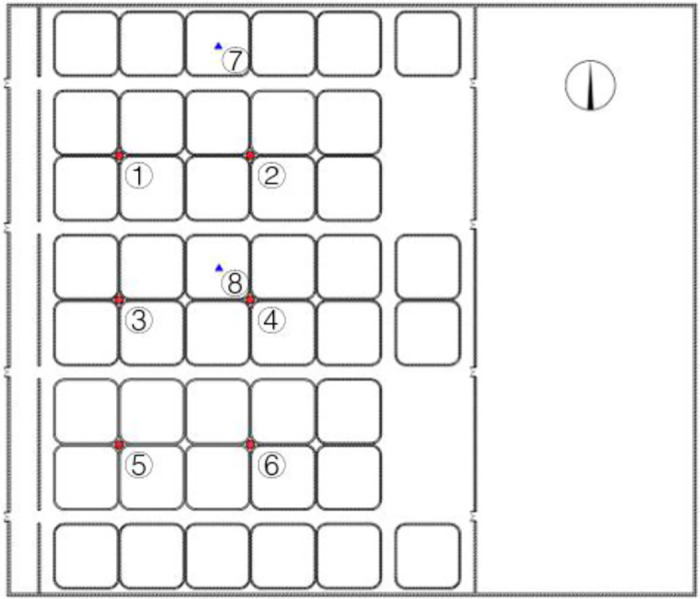
Workshop interior layout. **Note**: The red circle ⭕ indicates the location of the indoor temperature and humidity measuring point, and the blue triangle Δ indicates the water body temperature measuring point.

Fig 3 was the internal layout of the workshop. The air temperature and humidity measurement points are arranged at points 1–6 in the horizontal plane at a height of 1.5m from the ground in the aquaculture workshop. The water body temperature measuring points are arranged in the pool near the north wall at points 7 and 8, and the depth was located in the center of the pool. One measuring point (not marked in the figure) was set on the inner and outer surfaces of the roof respectively for temperature monitoring. Outdoors, a temperature and humidity meter and a light measuring instrument are arranged 3 meters south of the workshop to monitor the external environment.

### 3.3 Test instrument

The test instruments are shown in Table 3. The main test parameters are outdoor solar radiation, indoor and outdoor air temperature, water temperature of the breeding pond and heat flow of the wall.

**Table 3:** Test instrument.

Name	Model	Range	Accuracy
Light recorder	HOBO MX2202	0~167731 lux	±10%
Temperature and humidity loggers	HOBOUX100-011	-20~70°C	±0.2°C
Underwater temperature recorder	HOBO MX2201	-20~50°C	±0.5°C
Portable heat flow meter	JTDL-4	0~5 MW/m^2^	±4%

## 4. Verification and analysis

Since the light intensity on January 5, 2023 was weak, and the daily average outdoor temperature fluctuation was smaller than that of other experimental stages, the data of this day was selected as the verification data to evaluate the rationality of the numerical model. In order to verify the accuracy of the numerical calculation of the established workshop, the calculated data at the same position and at the same time as the measuring point are extracted and compared with the measured values.

**Fig 4:**
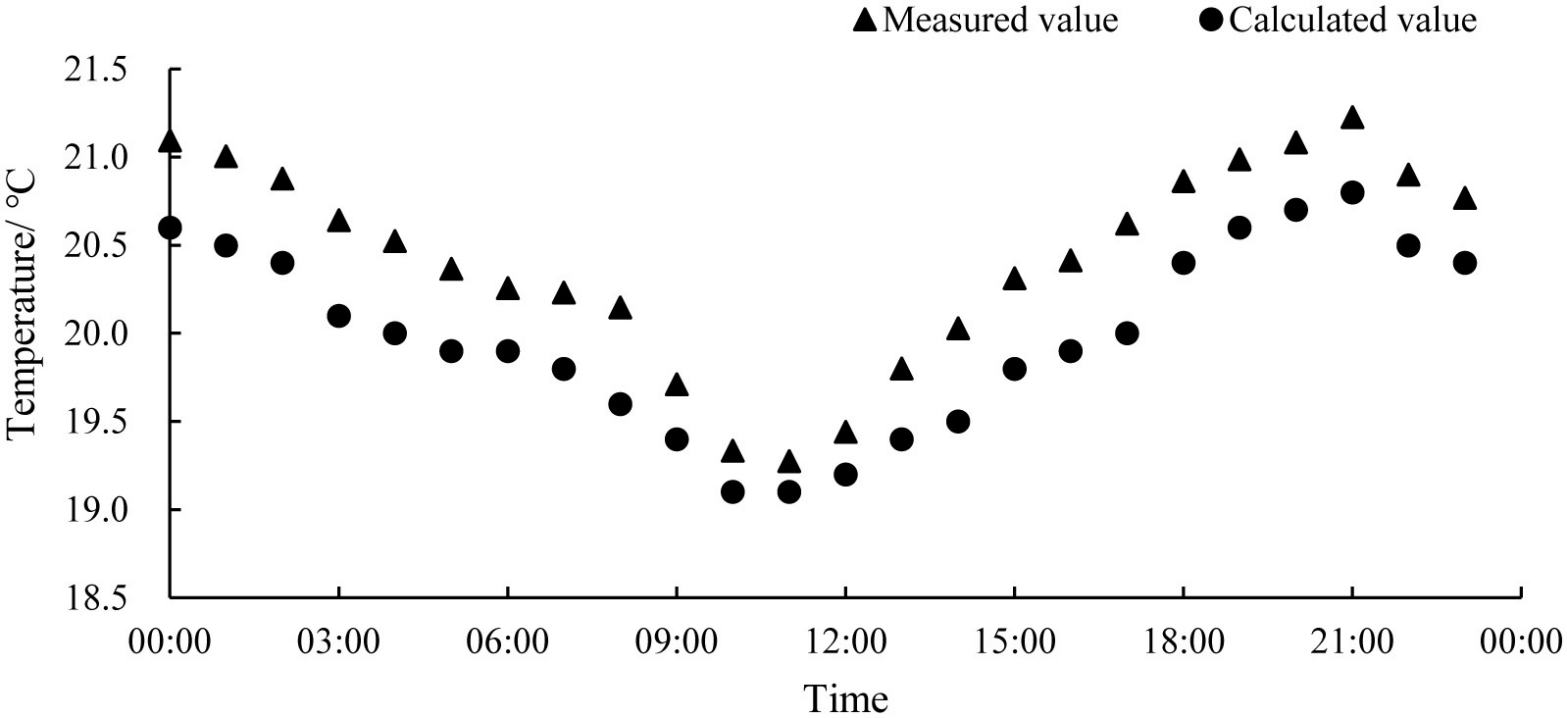
The measured and calculated values of the temperature in the workshop.

The measured and calculated values of the temperature in the industrialized aquaculture workshop are shown in Fig 4. On the whole, the calculation of indoor air by the numerical model was relatively accurate. The average absolute error was 0.43°C, the maximum error was 0.62°C, and the average relative error was 2.1%, so the calculation results are relatively reliable.

**Fig 5:**
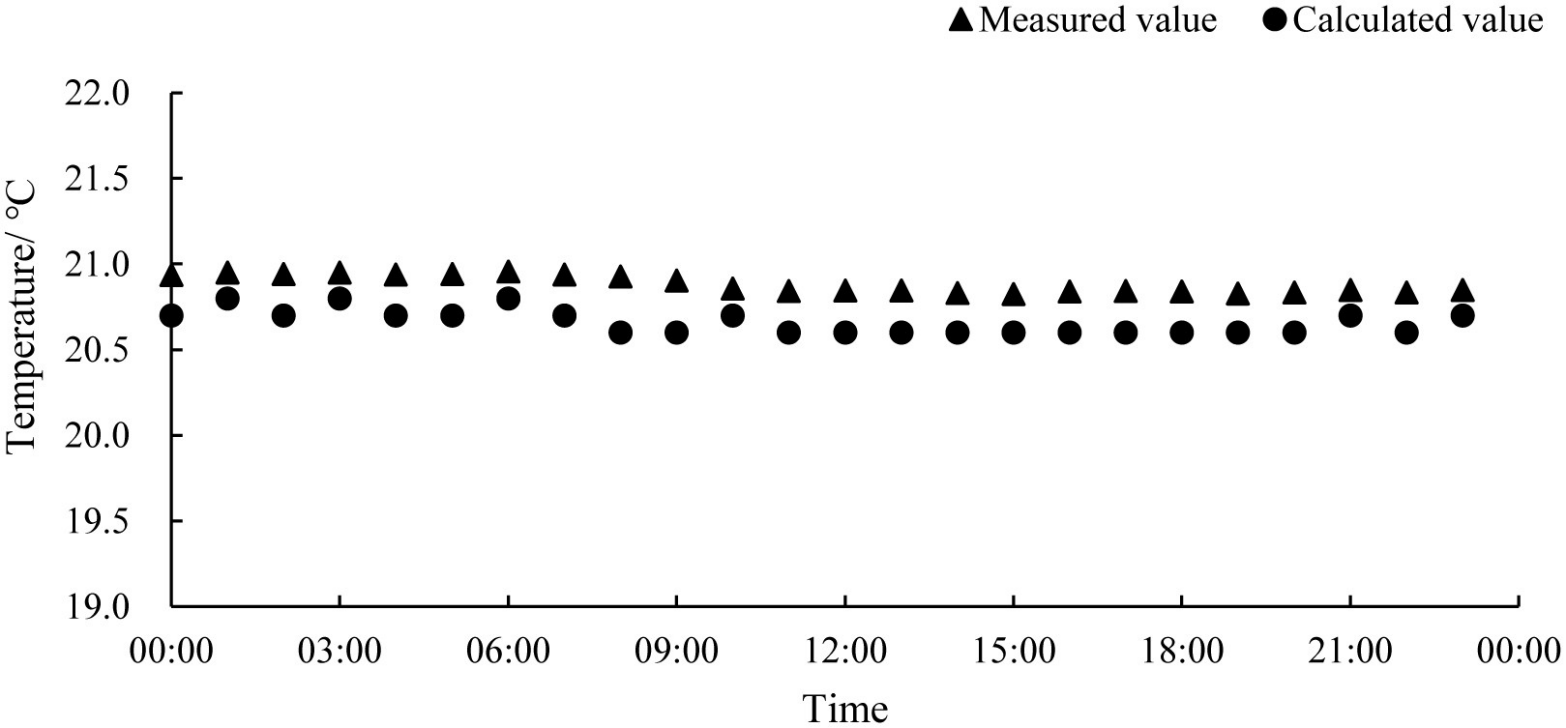
Measured and calculated values of water temperature in the workshop.

The measured and calculated values of water temperature in the industrialized aquaculture workshop are shown in Fig 5. On the whole, the calculation of the water temperature in the workshop by the numerical model was relatively accurate, with an average absolute error of 0.22°C, a maximum error of 0.33°C, and an average relative error of 1.1%. The calculation results of the water temperature of the aquaculture water body are highly accurate, mainly because the thermal inertia of the water was large, and the temperature of the water changes very little and remains basically stable.

**Fig 6:**
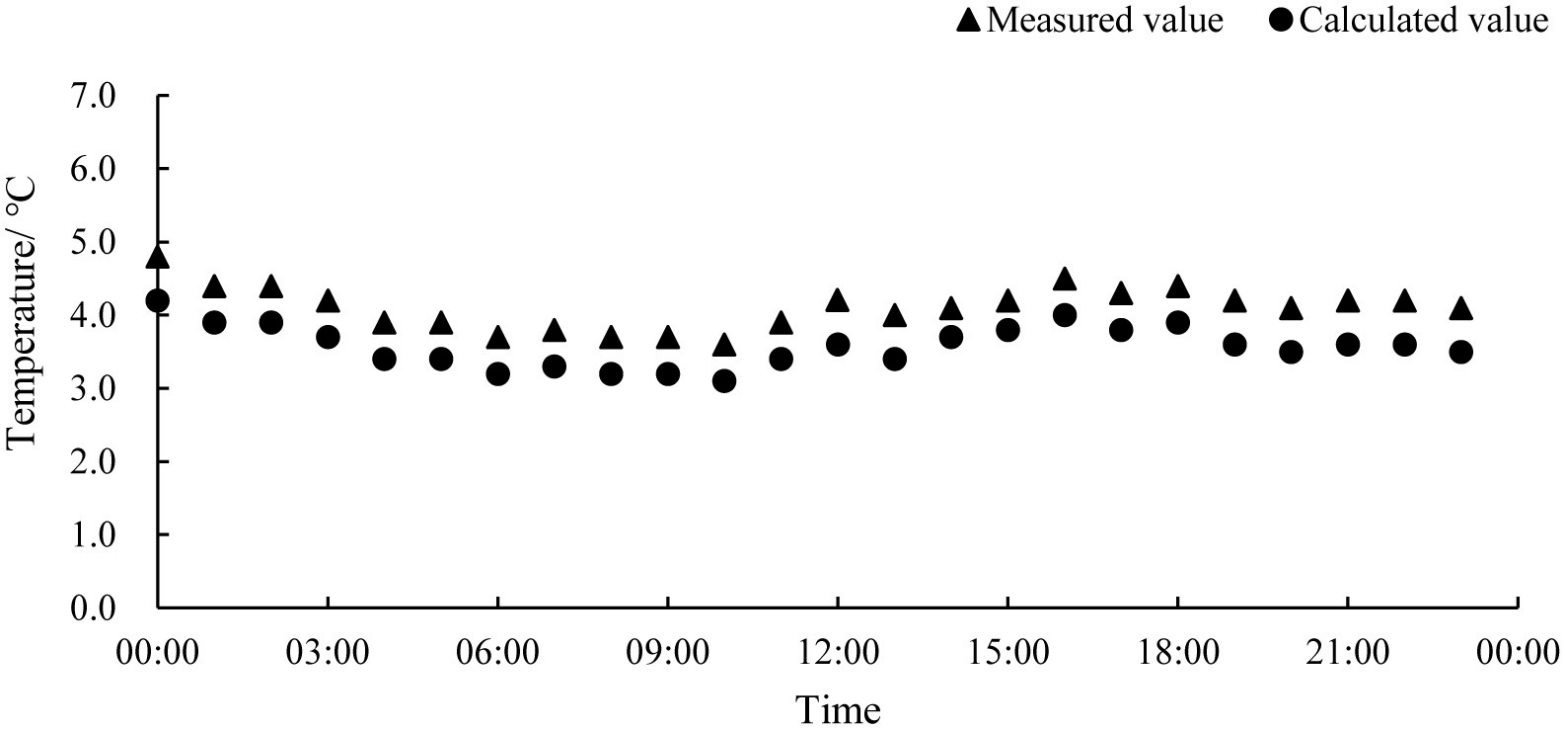
The measured and calculated values of the outer surface of the workshop roof.

The measured and calculated values of the outer surface temperature of the roof of the industrialized aquaculture workshop are shown in Fig 6. On the whole, the calculation of the outer surface temperature of the roof by the numerical model was relatively accurate. The temperature of the outer surface of the roof fluctuates greatly with external conditions, the average absolute error was 0.53°C, the maximum error was 0.6°C, and the average relative error was 9.8%. However, the calculation error was less than 10%, which can reflect the temperature change of the outer surface of the roof.

**Fig 7:**
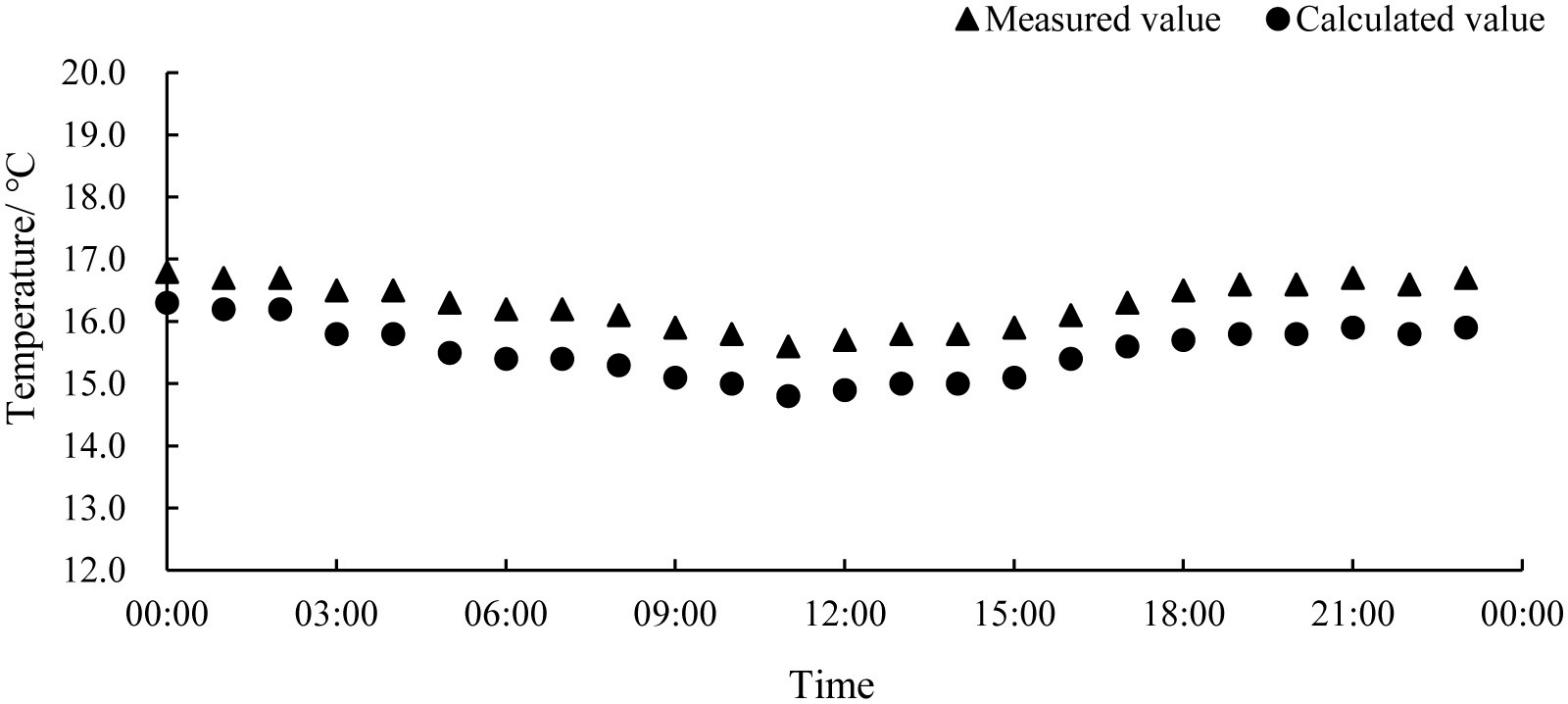
The measured and calculated values of the inner surface of the workshop roof.

The measured and calculated values of the inner surface temperature of the roof are shown in Fig 7. On the whole, the calculation of the inner surface temperature of the roof by the numerical model was relatively accurate, with an average relative error of 0.75°C, a maximum error of 0.8°C, and an average relative error of 2.1%. The calculation error of the inner surface temperature of the roof was small, mainly because the thermal environment inside the workshop was more stable, so the temperature fluctuation of the inner surface of the roof was reduced.

Based on the data analysis, the calculated temperature values were consistently lower than the measured temperature values, which may be attributed to the fact that the numerical model did not take into account the heat generated by the lighting and other equipment in the workshop. Additionally, although the calculation conditions simulated the external weather conditions on overcast days and did not consider solar radiation, in reality, solar radiation still contributes additional heat. These factors resulted in the systemic error observed in the numerical calculation results. In summary, the average relative error of this numerical model was within 5%, and the numerical calculation results can reflect the actual situation of the aquaculture workshop. so it can be used to analyze the distribution of its internal thermal environment.

**Fig 8:**
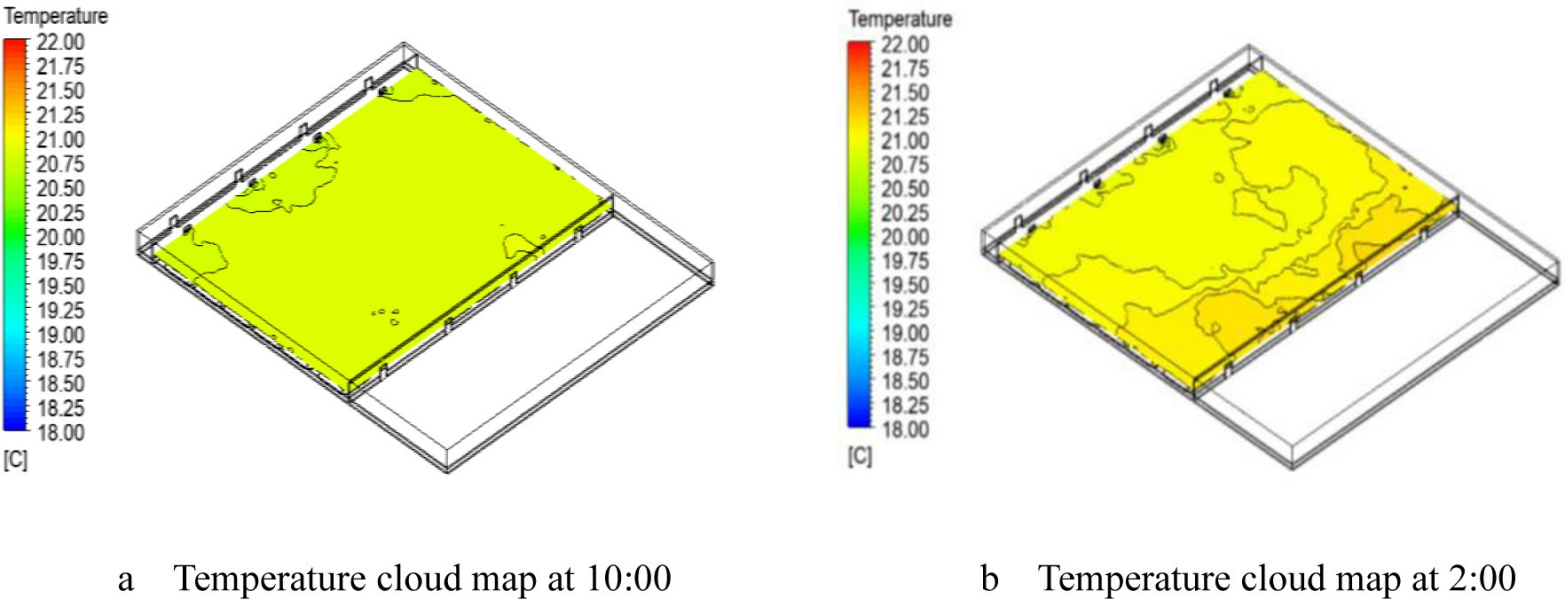
Temperature field at different times at 1.5m height on January 5, 2023. a. Temperature cloud map at 10:00, b. Temperature cloud map at 2:00.

Fig 8 shows the temperature distribution at the same height plane at different times. The lowest temperature of the day occurred at 10:00, while the highest temperature occurred at 2:00. It can be seen from the figure that the temperature in the workshop at 10:00 was lower than 20.5°C. The temperature in the workshop at 2:00 was slightly higher than the temperature at 10:00, mostly above 21°C. This was because the large specific heat capacity of water results in a small difference in temperature. The grouper is widely distributed in tropical and subtropical waters, with an optimal temperature range of 22–28°C and low cold tolerance [36, 37]. Experimental evidence suggests that the average optimal cultivation temperature for grouper is 26.32°C ± 0.62°C, and a cultivation temperature of 28°C may be the optimal choice for grouper hatcheries in the Asia-Pacific region [38]. The water temperature fluctuation range in the industrialized aquaculture workshop was: 19.7–21.1°C, which was slightly lower than the optimum growth temperature of the cultured grouper. The next step will be to optimize the existing workshop structure to meet the winter production needs of the aquaculture workshop.

## 5. Optimization

By changing the thermophysical characteristic parameters of the enclosure structure materials of the aquaculture workshop, the temperature field of the aquaculture pond in the workshop can be optimized, the thermal conductivity of the roof and the surrounding enclosure structure can be reduced, and the temperature field of the workshop can be improved. The optimization scheme was shown in Table 4.

**Table 4:** Optimization scheme table.

Optimization scheme	Optimization parameters
Scheme 1	The thermal conductivity of the envelope structure was reduced by 10%
Scheme 2	The thermal conductivity of the envelope structure was reduced by 20%

After optimization, the water temperature fluctuation range throughout the day of Scheme 1 was 19.8–21.5°C; the water temperature fluctuation range throughout the day of Scheme 2 was 20.4–21.8°C. However, even after optimization, the water temperature ranges for both Scheme 1 and Scheme 2 still fall below the optimum range for grouper fish, which was 22–28°C.

After calculating and optimizing the thermophysical parameters of the enclosure structure, it was found that the culture water temperature was still lower than the suitable growth water temperature range. If the thermophysical parameters of the enclosure structure continue to be reduced, the cost of workshop renovation will be too high, which was of little significance for actual production and application. Therefore, it was necessary to artificially increase the temperature of the aquaculture water in winter to ensure the normal production of the workshop.

In January, the temperature in Nantong City, Jiangsu Province, is relatively low, with an average temperature ranging from 0 to 8 degrees Celsius. Negative temperatures may occur during the night. The city experiences relatively high precipitation during this month, often accompanied by overcast and rainy weather. Sunshine duration is relatively low, with frequent cloudy and overcast conditions. Selected the meteorological parameter data in January of a typical year in Nantong area. According to the suitable temperature growth range of cultured grouper 22 ~ 28°C, three temperature gradients were set up, namely 22°C, 25°C and 28°C. The energy consumption of the aquaculture workshop was calculated and analyzed under different heating to constant temperature settings, so as to provide a theoretical basis for the winter heating strategy of the industrialized aquaculture workshop in this area. The formula for calculating the temperature of aquaculture water was as follows:

Q=cmΔt (8)

Where *Q* is the Heat absorbed (released), J/(kg⋅ °C); *c* is the Specific heat capacity, J/kg; *m* is the mass, kg; Δt is the Temperature difference, °C.

The solar radiation absorbed by the outer surface of the roof can be calculated using the following equation:

$$Q_{co-solar}=S\cdot A_{co}(1-\rho_{co-solar})\cdot \alpha_{co-solar}$$ (9)

Where *S* is the outdoor solar radiation intensity, W/m^2^; *A*_*co*_ is The area of the outer surface of the roof, m^2^; *ρ*_*co*−*solar*_ is the reflectivity of the outer surface of the roof to solar radiation, *ρ*_*co*−*solar*_ = 0.75; *α*_*co*−*solar*_ is the absorptivity of the outer surface of the roof to solar radiation, *α*_*co*−*solar*_ = 0.25.

### 5.1 Energy consumption analysis of industrialized aquaculture workshop (22°C)

When the indoor water temperature was set at a constant temperature of 22°C in January in winter, the heat loss in the workshop was shown in Table 5. Since the initial value of the water temperature during the test was 21.0°C, the water body needs to be heated by 1°C (replenishment energy was 9083 MJ). When the water temperature in the workshop was constant at 22°C, the total daily average heat loss of the workshop was 32,700 MJ, and the average daily solar radiation absorbed by the workshop was 5,372MJ, so the daily supplementary energy to the workshop was 27,328 MJ. Therefore, during the wintering period (January), if the water temperature in the industrialized aquaculture workshop was kept at 22°C, the required energy consumption was 8.56×10^5^ MJ, which was about the energy released by the complete combustion of 29.3t standard coal.

**Table 5:** Heat loss in constant temperature aquaculture workshop (22°C).

	Roof heat loss (MJ/d)	Underground heat transfer loss (MJ/d)	Air heat exchange loss (MJ/d)
Minimum	14,288	13,270	2,126
Average	15,463	13,270	3,967
Maximum	16,875	13,270	5,456

### 5.2 Energy consumption analysis of industrialized aquaculture workshop (25°C)

When the indoor water temperature was set to a constant temperature of 25°C in January in winter, the water body needs to be heated to 4°C (replenishment energy was 36,332 MJ). Calculation of the heat loss in the workshop where the water temperature was constant at 25°C in January was shown in Table 6. When the water temperature in the workshop was constant at 25°C, the total daily average heat loss of the workshop was 37,294 MJ, and the average daily solar radiation absorbed by the workshop was 5,372 MJ, so the daily supplementary energy to the workshop was 31,922 MJ. Therefore, during the winter, if the water temperature in the industrialized aquaculture workshop was kept at 25°C, the required energy consumption was 1.02×10^6^ MJ, which was about the energy released by the complete combustion of 35.1t standard coal.

**Table 6:** Heat loss in constant temperature aquaculture workshop (25°C).

	Roof heat loss (MJ/d)	Underground heat transfer loss (MJ/d)	Air heat exchange loss (MJ/d)
Minimum	16,354	15,975	2,376
Average	17,374	15,975	3,945
Maximum	18,876	15,975	5,675

### 5.3 Energy consumption analysis of industrialized aquaculture workshop (28°C)

When the indoor water temperature was set at a constant temperature of 28°C in January in winter, the water body needs to be heated by 7°C (replenishment energy was 6,3581 MJ). Calculate the heat loss in the workshop with the water temperature constant at 28°C in January, as shown in Table 7. When the water temperature in the workshop was constant at 28°C, the daily average total heat loss of the workshop was 42,778 MJ, and the average daily solar radiation absorbed by the workshop was 5,372 MJ, so the daily supplementary energy to the workshop was 37,406 MJ. Therefore, during the winter, if the water temperature in the industrialized aquaculture workshop was kept at 28°C, the required energy consumption was 1.22×10^6^ MJ, which was about the energy released by the complete combustion of 41.8t standard coal.

**Table 7:** Heat loss in constant temperature aquaculture workshop (28°C).

	Roof heat loss (MJ/d)	Underground heat transfer loss (MJ/d)	Air heat exchange loss (MJ/d)
Minimum	17367	18,623	2,567
Average	19,568	18,623	4,587
Maximum	21,528	18,623	6,739

## 6. Discussion

The current modeling primarily focuses on the heat loss of maintenance structures in aquaculture facilities during low-temperature climates in winter. Therefore, the latent heat of water evaporation within the facility has not been taken into account in the calculations [39]. Additionally, some secondary factors have been simplified during the calculation process, such as ignoring the effects of heat generated by lamps and metabolic heat produced by organisms in the pool.

It can be seen from the thermal image that the temperature of the water area near the inner wall was higher than that of other areas. We had discovered significant energy losses within the interior of the aquaculture workshop, particularly at maintenance structures directly exposed to the external environment. The limited insulation properties of the external maintenance structure materials had resulted in substantial heat loss due to high temperature differentials [40]. In order to reduce heat loss within the aquaculture facilities, we had appropriately enhanced the insulation properties of the external maintenance structure materials. However, the results still failed to meet the minimum water temperature requirements for winter production in the aquaculture facilities. As a result, artificial heating was required in the region during the winter production, leading to increased production costs. Therefore, we further utilized a numerical model to calculate the heat losses of various components of the enclosure structures under different water temperature conditions.

The analysis was of the optimization data concluded that additional heat supplementation was needed to ensure the normal production of the industrialized aquaculture workshop in winter. Data processing was shown in Table 8. In January, the production temperature of the workshop was kept between 22–25°C and the heating efficiency was 5.5×10^4^ MJ/ °C; and the temperature replenishment efficiency of the workshop production temperature kept between 25–28°C was 6.7×10^4^ MJ/ °C. From the above analysis, it can be seen that keeping the temperature at 22–25°C was more efficient, and producers can reasonably plan breeding varieties and overwintering production plans according to needs.

**Table 8:** Supplementary calorie calculation table.

Constant temperature setting value	Need to add energy value
22°C	8.56×10^5^ MJ
25°C	1.02×10^6^ MJ
28°C	1.22×10^6^ MJ

## 7. Conclusion

The temperature field distribution in the industrialized aquaculture workshop was calculated and verified using CFD technology. The results showed that the average relative error between the measured and calculated values of the indoor air temperature, water temperature, and roof inner surface temperature in the industrialized aquaculture workshop was within 2.5%. The numerical model can be used to analyze and study the thermal environment in the industrialized aquaculture workshop.

After analyzing the temperature distribution within the aquaculture facility, it was found that heat loss primarily occurs at the maintenance structures directly exposed to the external environment. The temperature field in the workshop was optimized by improving the thermal performance parameters of the enclosure structure of the industrialized aquaculture workshop. The results show that by changing the thermal performance parameters of the enclosure structure, the minimum standard for overwintering production of aquaculture water temperature cannot be achieved, so artificial warming of the aquaculture water was required.

By calculating the average heat consumed when heating to a constant temperature and the heat supplied and input, it was obtained: When maintaining the workshop temperature at 22°C in January, an additional 8.56×10^5^ MJ energy needs to be input; when maintaining the workshop temperature at 25°C, an additional 1.02×10^6^ MJ energy needs to be input; when maintaining the workshop temperature at 28°C, an additional 1.22×10^6^ MJ energy needs to be input. And it was concluded that the heating efficiency of industrialized aquaculture workshop was higher when the temperature of overwintering production was maintained at 22–25°C. This conclusion can provide theoretical basis and application reference for industrialized aquaculture in winter.

## Supporting information

S1 File (XLSX)
